# Optimization of the Isolation and Purification of Formononetin from *Lycopus lucidus* Turcz. var. *hirtus* Regel, Structural Identification, and Its In Vitro Anti-Inhibitory Effects in TNF-α-Induced HFLS-RA Cells

**DOI:** 10.3390/plants15172654

**Published:** 2026-08-29

**Authors:** Feiran Hu, Wenzhao Zhou, Nong Zhou, Lanlan Wan, Linyun Pu, Caiyun Fu, Hua Zhang, Junsheng Qi

**Affiliations:** 1 College of Biology and Food Engineering, Chongqing Sanxia University of Science and Technology, Chongqing 404120, China; 2 Chongqing Resources Branch, National Engineering Research Center for Modernization of Traditional Chinese Medicine, Chongqing 404500, China; 3 Green Planting and Deep Processing for the Three Gorges Reservoir Region Indigenous Medicinal Herbs of Chongqing Engineering Research Center, Chongqing Key Laboratory of Development and Utilization of Genuine Medicinal in Three Gorges Reservoir Area, Chongqing 404120, China

**Keywords:** *Lycopus lucidus* Turcz. var. *hirtus* Regel, flavonoids, formononetin, rheumatoid arthritis, synoviocytes

## Abstract

*Lycopus lucidus* Turcz. var. *hirtus* Regel (*L. lucidus* var. *hirtus*) is a medicinal herb traditionally used in Chinese folk medicine for the treatment of inflammatory disorders, joint pain, and rheumatoid arthritis (RA)-like symptoms. However, systematic research on the bioactive flavonoids of this herb in RA-related cellular models is limited. The aim of this study was to isolate the main flavonoid from this herb and evaluate its in vitro anti-inhibitory effects in TNF-α-induced human RA fibroblast-like synoviocytes (HFLS-RA). Flavonoids were extracted, purified, and isolated using chromatographic techniques. The structure was elucidated by spectroscopic analysis. The effects of the isolated compound were assessed in a TNF-α-induced HFLS-RA cell model by evaluating pro-inflammatory cytokine secretion and cell proliferation/metabolic activity. A main flavonoid was identified as formononetin. Formononetin exhibited antioxidant activity and inhibited TNF-α-provoked inflammatory responses in HFLS-RA cells under cell-safe concentrations, as evidenced by reduced secretion of IL-6, IL-8, and IL-1β and suppression of TNF-α-induced hyperproliferation. In conclusion, formononetin is a major flavonoid constituent of *L. lucidus* var. *hirtus*, and these findings provide preliminary in vitro cellular evidence that formononetin exerts in vitro anti-inflammatory inhibitory activity on TNF-α-activated human RA fibroblast-like synoviocytes. Further in vivo and mechanistic studies are required to explore its potential for anti-RA-related pharmacological application.

## 1. Introduction

*Lycopus lucidus* Turcz. var. *hirtus* Regel is a perennial herb belonging to the genus *Lycopus* of the family Lamiaceae. It is a variety of *Lycopus lucidus* Turcz. and is valued for its medicinal underground rhizome. Morphologically, it has creeping rhizomes with often swollen, moniliform or fusiform nodes; erect, quadrangular stems with dense hirsute hairs at the nodes; opposite, subsessile, lanceolate or narrowly lanceolate leaves with acuminate apex, attenuate base, sharply serrate margins, and short hirsute hairs or glandular dots on both surfaces. Its verticillasters are axillary and densely flowered, with campanulate calyx, bilabiate white or pale purple corolla, and brown ovoid-tetragonal nutlets. Due to its morphological resemblance and comparable nutritional profile to ginseng, it is also commonly referred to by various aliases such as “Chongcaoshen”, “Dicangzi”, and “Caoshican” [1]. In traditional Chinese folk medicine, the rhizome of *L. lucidus* var. *hirtus* has long been used for alleviating inflammatory conditions, relieving joint swelling and pain, and ameliorating RA-like clinical manifestations, with a long history of application in the field of ethnopharmacology for rheumatic and immune-related disorders. Currently, related products derived from this herb are primarily developed as dietary supplements and herbal health products in the regional market, while evidence-based pharmaceutical development is still in the preliminary research stage. Modern phytochemical research indicates that *L. lucidus* var. *hirtus* contains a wide range of bioactive constituents, including polyphenols (among which flavonoids are a prominent subclass), triterpenoids, polysaccharides, and steroidal compounds [2,3,4]. Phytochemical investigations on this Lamiaceae taxon have identified major secondary metabolites including phenolic acids (rosmarinic acid, caffeic acid, chlorogenic acid and their derivatives), flavonoids (luteolin, apigenin and their glycosides), triterpenoids (ursolic and oleanolic acids), and essential oil constituents. Correspondingly, this herb exhibits multiple pharmacological properties such as hepatoprotective, antioxidant, antitumor, hypoglycemic, hypolipidemic, immunomodulatory, and anti-rheumatic-relevant bioactivities [5,6,7].

Synovial inflammation in rheumatoid arthritis (RA) involves a complex cascade of interactions between immune and non-immune cell subsets [8]. A 2021 report estimated the global prevalence of RA to be approximately 0.46% [9], with women exhibiting a 2–3 times higher incidence rate than men [10]. The Global Burden of Disease 2021 collaborators for RA projected that the number of RA patients could reach 31.7 million by 2050 [11]. From a socioeconomic perspective, the substantial economic burden associated with RA has historically been underestimated [12]. In vitro research on RA frequently employs tumor necrosis factor-alpha (TNF-α)-induced human rheumatoid arthritis fibroblast-like synoviocytes (HFLS-RA) as a model system [13]. Current clinical pharmacotherapy for RA primarily relies on non-steroidal anti-inflammatory drugs (NSAIDs), disease-modifying antirheumatic drugs (DMARDs), biologic and targeted synthetic DMARDs, as well as glucocorticoids [14]. However, the use of these agents is often accompanied by significant adverse effects, including hepatotoxicity [15], gastrointestinal complications [16], and hypersensitivity reactions [17]. Consequently, there is a pressing need to develop safer and more effective therapeutic strategies for RA management.

Notably, flavonoids have emerged as compounds holding potential for anti-RA-associated pharmacological investigation. Studies suggest a strong correlation between the in vitro anti-inflammatory properties of flavonoids and their specific chemical structures. For instance, a 3′,4′-dihydroxy substitution pattern is known to enhance anti-inflammatory potency. The presence of a C2-C3 double bond, alongside hydroxyl groups at the C-4, C-3, and C-5 positions, is favorable for inhibiting pro-inflammatory cytokine production. Furthermore, the combination of a C2-C3 double bond with a 5,7-dihydroxy configuration contributes to the inhibition of nitric oxide (NO) generation, thereby augmenting anti-RA effects [18]. Wang Hongli et al. [19] provided in vivo evidence demonstrating the therapeutic efficacy of total flavonoids from *Taxillus chinensis* against RA. Zhang Liguo et al. [20] compared the anti-inflammatory, immunomodulatory, and osteocyte-repair effects of different flavonoid-enriched fractions from traditional Chinese medicines, offering a rationale for screening effective fractions as candidate agents for rheumatic diseases. Among these, the total flavonoids from *L. lucidus* var. *hirtus* were shown to possess anti-rheumatic-related bioactivity in the study by Jiang Chun-yang [7]; however, the specific key monomeric compounds responsible for this efficacy remain unidentified.

With regard to flavonoids, formononetin, a classic isoflavone compound widely distributed in leguminous medicinal plants, has been extensively documented for its anti-inflammatory and arthritis-relevant pharmacological activities. Preclinical studies in collagen-induced arthritis (CIA) mouse models have confirmed that formononetin can reduce the arthritis index, alleviate joint swelling, inhibit synovial inflammatory cell infiltration and cartilage damage, and downregulate the expression of pro-inflammatory cytokines such as TNF-α, IL-1β and IL-6, effects that have been associated with inhibition of the NF-κB signaling pathway [21,22]. However, these mechanistic findings were mostly obtained from animal and macrophage models, and the direct effects of formononetin on human RA fibroblast-like synoviocytes, the core effector cells driving synovial hyperplasia and joint destruction, remain insufficiently characterized.

Therefore, this study was designed to optimize the extraction and enrichment procedure of total flavonoids from *L. lucidus* var. *hirtus*, identify formononetin as the principal bioactive flavonoid in the purified fraction, and further evaluate its antioxidant and in vitro anti-inhibitory activity on TNF-α-induced HFLS-RA cells. By characterizing this specific bioactive monomer and its effect, this work clarifies the pharmacodynamic material basis of this ethnic medicinal herb beyond the previously reported total flavonoid level, and provides experimental support for understanding its arthritis-relevant anti-inflammatory potential. This research will not only contribute to a deeper understanding of in vitro anti-RA-associated effects of *L. lucidus* var. *hirtus* flavonoids and enhance the resource utilization of this herb, but also provide a theoretical foundation for the development of novel anti-RA natural products and their subsequent scale-up production.

## 2. Materials and Methods

### 2.1. Materials

The rhizomes of *L. lucidus* var. *hirtus* were collected from Wanzhou, Chongqing, and authenticated by Professor Zhou Nong of Chongqing Three Gorges University. The samples were identified as the dried rhizomes of *L. lucidus* var. *hirtus* (Lamiaceae) based on their morphological characteristics, such as distinct nodes with scale leaves and sparse fibrous roots, and a cylindrical, enlarged apex. A voucher specimen (No. S221-20240912) has been deposited in the Medicinal Plant Herbarium of Chongqing Three Gorges University, Wanzhou, Chongqing, China.

Rutin standard (purity ≥ 99%), hydroxychloroquine, cellulase, hemicellulase, pectinase, AB-8 macroporous resin, formononetin (Batch No. st002601-16523, purity ≥ 98%), were purchased from Shanghai Yuanye Bio-Technology Co., Ltd., Shanghai, China. The HFLS-RA cell line was obtained from Huatuo Bio-Technology Co., Ltd., Guangzhou, China. DMEM-H medium, fetal bovine serum (FBS), dimethyl sulfoxide (DMSO), penicillin-streptomycin solution, phosphate-buffered saline (PBS), and 0.25% trypsin-EDTA digestion solution were procured from Wuhan Serviebio Technology Co., Ltd., Wuhan, China. TNF-α was sourced from Beyinle Bio-Technology Co., Ltd. (Cat. No. HTX1835), Beijing, China. Enzyme-linked immunosorbent assay (ELISA) kits for IL-1β, IL-6, and IL-8 were obtained from Beijing Solarbio Science & Technology Co., Ltd., Beijing, China. Dextran gel Sephadex LH-20 was purchased from Shanghai Enzyme-linked Biotechnology Co., Ltd., Shanghai, China. D101 macroporous resin was acquired from Ica Bio-Technology Co., Ltd., Shanghai, China. D4020 macroporous resin was supplied by Zhengzhou Hecheng New Material Technology Co., Ltd., Shanghai, China. H103 and NKA-9 macroporous resins were both purchased from Hangzhou Huiying Instrument Co., Ltd., Hangzhou, China. Methanol (HPLC grade) and sodium nitrite were bought from Chengdu Kelong Chemical Co., Ltd., Chengdu, China. Absolute ethanol, n-butanol, and trichloromethane were obtained from Chongqing Chuandong Chemical (Group) Co., Ltd., Chongqing, China. Hydrochloric acid was purchased from Chengdu Kelong Chemical Reagent Factory, Chengdu, China. Aluminum nitrate was sourced from Aladdin Reagent (Shanghai) Co., Ltd., Shanghai, China. Sodium hydroxide was procured from Shanghai Yien Chemical Technology Co., Ltd., Shanghai, China.Dipotassium hydrogen phosphate was acquired from Xilong Scientific Co., Ltd., Shantou, China. All chemical reagents used were of analytical grade, and distilled water was used throughout the experiments.

### 2.2. Extraction of Crude Extract for Flavonoid Enrichment from Lycopus lucidus Turcz. var. hirtus Regel

#### 2.2.1. Ethanol Reflux Extraction of Crude Herbal Extract

The dried rhizome was pulverized and sieved through a 40-mesh screen. Powder samples were reflux-extracted in a thermostatically controlled water bath to obtain a crude multi-component ethanolic extract for subsequent flavonoid enrichment and purification. Following the method described in reference [23] and keeping other conditions constant, single-factor experiments were conducted to investigate the effects of ethanol concentration (55, 65, 75, 85, and 95%), reflux time (1, 2, 3, 4, and 5 h), number of extraction cycles (1, 2, 3, 4, and 5), and solid–liquid ratio (1:10, 1:15, 1:20, 1:25, and 1:30 g/mL). All dilution-induced volume changes were fully incorporated when calculating extraction-related indices.

#### 2.2.2. Determination of Total Flavonoid Content

The total flavonoid content was determined using the aluminum nitrate colorimetric method as described by Pękal and Pyrzynska [24] with slight modifications. Briefly, rutin standard solutions (0.3, 0.6, 0.9, 1.2, 1.5, 1.8, 2.1, 2.4, and 2.7 mL) were accurately pipetted and diluted to 10 mL with 60% ethanol. To each solution, 0.3 mL of 5% NaNO_2_ solution was added. After standing for 3 min, 0.3 mL of 10% Al (NO_3_)_3_ solution was added and allowed to react for 4 min. Then, 4 mL of 1 M NaOH solution was added, and the volume was finally adjusted to 10 mL with 60% ethanol. After 11 min of reaction, the absorbance was measured at 510 nm using a visible spectrophotometer (V-1100D, Shanghai Mapada Instruments Co., Ltd., Shanghai, China) against a blank of anhydrous ethanol. A standard calibration curve was plotted with concentration (mg/mL) as the x-axis and absorbance as the y-axis. The regression equation was y = 1.1973x + 0.0089 (R^2^ = 0.9996), indicating a good linear relationship within the concentration range of 0.01 to 1.0 mg/mL. The total flavonoid content in the extracts was calculated based on this calibrated standard curve and expressed as rutin equivalents.

The extraction yield of total flavonoids was calculated as follows:

$$Y_{TF}=\frac{m_{TF}}{m_{raw}}\times 100\%$$

In this formula, $Y_{TF}$ represents the extraction yield (%) of total flavonoids; $m_{TF}$ stands for the mass of total flavonoids quantified as rutin equivalents, with the unit of milligram (mg); $m_{raw}$ denotes the dry mass of raw medicinal herb powder, also measured in mg.

Notably, this colorimetric method may cross-react with co-existing phenolic compounds and reducing sugars in the sample matrix; thus, the results reflect the relative enrichment of flavonoid-like substances rather than the absolute mass of pure flavonoids.

### 2.3. Purification of Flavonoids from Lycopus lucidus Turcz. var. hirtus Regel Using Macroporous Resin

#### 2.3.1. Selection of Macroporous Resin

Prior to column packing, five macroporous resins (AB-8, D101, D4020, H103, and NKA-9; their physicochemical properties are listed in Table 1) were pretreated and, when necessary, regenerated following the method of Ye et al. [25]. The pretreatment consisted of soaking the resins in anhydrous ethanol for 24 h, washing with distilled water, sequential treatment with 5% HCl and 2% NaOH solutions overnight, and a final rinse with distilled water until a neutral pH was reached. The resins were subsequently stored in ethanol. For regeneration, the exhausted resins were eluted with a large volume of 95% ethanol until the eluate became colorless, and then thoroughly washed with water.

After pretreatment, 2.0 g of each resin (dry weight) was precisely weighed using an analytical balance and placed into a 100 mL conical flask. Then, 20 mL of the crude flavonoid extract was added. The flasks were shaken on an orbital shaker at 120 rpm and 25 °C for 24 h for adsorption. After adsorption, the supernatant was collected to determine its absorbance and calculate the flavonoid concentration. The resin was filtered, washed with distilled water until the washings were colorless, and the surface moisture was removed with filter paper. The resin was then transferred to a new dry conical flask, and 20 mL of 95% ethanol was added for desorption under the same shaking conditions for 24 h. The flavonoid concentration in the desorption solution was determined. The adsorption capacity (Q_0_, mg/g), adsorption ratio (Q_1_, %), and desorption ratio (Q_2_, %) were calculated using the following equations to evaluate resin performance:

(1) $$Q_{0}=\frac{\left(C_{0}-C_{1}\right)\times V_{1}}{m},$$

(2) $$Q_{1}=\frac{C_{0}-C_{1}}{C_{0}}\times 100\%,$$

(3) $$Q_{2}=\frac{C_{2}\times V_{2}}{Q_{0}\times m}\times 100\%,$$

where m is the mass of the resin (g); C_0_, C_1_, and C_2_ are the flavonoid concentrations (mg/mL) in the initial solution, the solution after adsorption equilibrium, and the desorption solution, respectively; and V_1_ and V_2_ are the volumes (mL) of the adsorption and desorption solutions, respectively.

#### 2.3.2. Optimization of Purification Conditions via Single-Factor and Orthogonal Tests

Following resin selection, the purification process parameters were further optimized. First, the optimal sample concentration was determined through static adsorption–desorption experiments. Then, dynamic adsorption–desorption single-factor experiments were conducted to investigate the effects of ethanol concentration in the eluent (10, 30, 50, 70, and 95%), sample pH (3, 4, 5, 6, and 7), and elution flow rate (2, 3, 4, 5, and 6 mL/min) on the purification efficiency.

To systematically identify the optimal purification conditions, an L_9_(3^4^) orthogonal array design was implemented based on the results of the single-factor experiments. Three key factors were evaluated, each at three levels: sample pH (4, 5, 6), eluent ethanol concentration (50, 70, 90%), and elution flow rate (2, 3, 4 mL/min). The flavonoid recovery yield was used as the response variable to assess the influence of each factor.

### 2.4. Determination of Natural Ratio, Isolation, and Identification of Individual Flavonoids from Lycopus lucidus Turcz. var. hirtus Regel

#### 2.4.1. Determination of the Natural Ratio of Flavonoid Compounds

To determine the relative proportion of individual flavonoids, the purified flavonoid mixture was dissolved in methanol and analyzed by high-performance liquid chromatography (HPLC). The analysis was performed on a BioLogic DuoFlow Pathfinder 80 System (Bio-Rad Laboratories, Inc., Hercules, CA, USA) equipped with a Venusil XBP C-18 column (4.6 × 250 mm, 5 µm; Agela Technologies, Tianjin, China). The mobile phase consisted of methanol (A) and 0.4% (*v*/*v*) phosphoric acid aqueous solution (B) with the following gradient elution program: 0 min, 20% A; 10 min, 35% A; 25 min, 55% A; 30 min, 80% A; 35 min, 80% A; 40 min, 20% A. The total run time was 45 min per injection, including a 5 min column re-equilibration step to restore initial conditions and ensure stable retention across consecutive runs. The flow rate was 1.0 mL/min, the injection volume was 10 µL, the detection wavelength was set at 360 nm, and the column temperature was maintained at 20 °C [26,27].

Method validation was conducted, and the results demonstrated good reliability of the analytical method: the relative standard deviation (RSD) of peak area was less than 2.1% for six consecutive injections, indicating satisfactory instrument precision; the peak area RSD remained below 2.8% when the sample solution was analyzed at 0, 2, 4, 8, 12 and 24 h at room temperature, confirming acceptable sample stability within 24 h; for six independently prepared parallel samples, the RSD of the peak area ratio between the two main components was less than 3.0%, verifying favorable method repeatability; in addition, the RSD of retention time for each of the seven main peaks was below 1.2% across six consecutive injections, showing stable retention performance. Linearity, limits of detection/quantification, and matrix effects were not assessed in the present study.

#### 2.4.2. Isolation and Identification of Individual Flavonoids

To obtain high-purity flavonoids for structural identification, a two-step chromatographic separation strategy combining Sephadex LH-20 medium-pressure column chromatography (MPLC) and reversed-phase preparative HPLC was employed, using 10 g of crude extract as the starting material. Each separation step was monitored by analytical HPLC to ensure progressive enrichment of target components.

MPLC on Sephadex LH-20: Dry Sephadex LH-20 gel was soaked in the starting mobile phase (methanol–water, 20:80, *v*/*v*) overnight at room temperature with intermittent gentle stirring to ensure complete swelling. The column (1.5 × 50 cm) was wet-packed and equilibrated with the same starting mobile phase at a flow rate of 1 mL/min for 3 column volumes. The sample, dissolved and filtered through a 0.45 µm membrane, was loaded onto the column using the wet loading method. Elution was performed using a linear gradient from 20% to 100% methanol over 300 min at a flow rate of 1.0 mL/min. The eluate was monitored at 290 nm, and fractions were automatically collected (10 mL/tube) using a fraction collector [28,29].

The separation on Sephadex LH-20 is governed by a combination of size-exclusion effect and reversed-phase adsorption mechanism. The porous gel matrix separates components by molecular size, while the hydroxypropyl-modified gel skeleton exerts hydrophobic interactions with flavonoid aromatic rings. Gradient elution with increasing methanol proportion weakens hydrophobic binding and achieves sequential elution of components with different polarities. Fractions eluting between 220 and 270 min were combined, concentrated by rotary evaporation, and lyophilized to obtain a preliminarily purified powder, which was verified as the flavonoid-enriched fraction (Fraction C) with the highest target content by HPLC analysis.

HPLC purification in repeated-injection mode: The powder obtained from the previous step was further purified by HPLC using an analytical Venusil XBP C-18 column (4.6 × 150 mm, 5 µm; Agela Technologies, Tianjin, China). Isocratic elution with methanol–water (20:80, *v*/*v*) was applied at a flow rate of 0.3 mL/min. The injection volume was 10 µL, detection was performed at 290 nm, and the column temperature was maintained at 20 °C [30,31]. A total of 45 injections were performed. The isocratic elution condition with 20% methanol was optimized to achieve baseline separation between the target compound and coexisting impurities. The major target peak with a retention window of 13–14 min was collected, combined, concentrated by rotary evaporation and lyophilized. The purity of the isolated monomer was verified by HPLC under the conditions described in Section 2.4.1. The compound showed a single symmetrical peak on the chromatogram, with purity sufficient for subsequent structural identification and bioactivity assays.

A hyphenated strategy of “stepwise chromatographic purification coupled with multi-spectroscopic elucidation” was adopted for structural identification. Chromatographic techniques served to provide high-purity compound verified as single symmetrical peak by analytical HPLC, which guaranteed that all subsequent spectroscopic signals originated exclusively from the target compound without impurity interference.

Subsequently, structural elucidation was performed by integrating data from ultraviolet–visible spectroscopy (SHIMADZU UV-2401PC spectrophotometer, Shimadzu, Kyoto, Japan), Fourier transform infrared spectroscopy (BRUKER VERTEX 70 spectrometer; Bruker Corporation, Billerica, MA, USA), high-resolution mass spectrometry (Q-Exactive Focus quadrupole-Orbitrap mass spectrometer; Thermo Fisher Scientific, Waltham, MA, USA), and nuclear magnetic resonance spectroscopy (Bruker DRX-500 spectrometer; Bruker Corporation, Billerica, MA, USA) [32]. UV spectroscopy was applied to determine the flavonoid parent nucleus type via characteristic absorption bands; IR spectroscopy identified core functional groups including hydroxyl, carbonyl and aromatic ring; HR-MS provided accurate molecular weight and deduced molecular formula; NMR spectroscopy achieved full assignment of carbon and hydrogen skeletons. Based on comprehensive spectroscopic analysis and comparison with published reference data, the isolated monomer was identified as formononetin. All original spectra are provided in the Supplementary Materials.

### 2.5. Antioxidant Activity of Formononetin and Its Effects on TNF-α-Induced HFLS-RA Cells

#### 2.5.1. In Vitro Antioxidant Activity Assays

The antioxidant activity of the isolated flavonoid, formononetin, was evaluated by determining its DPPH and ABTS radical scavenging capacities, as well as its ferric reducing antioxidant power (FRAP). The half-maximal inhibitory concentration (IC_50_) was calculated for each assay, expressed in both mass concentration (μg/mL) and molar concentration (μmol/L) [33]. The IC_50_ value was calculated using the following formula:

$$Y=Bottom+\frac{Top-Bottom}{1+10^{\left(\log{IC}_{50}-X\right)\times HillSlope}}$$

where Y represents the percentage scavenging rate, X is the logarithm of the sample concentration, Bottom and Top denote the lower and upper plateaus of the fitted curve, respectively, and HillSlope describes the steepness of the curve. The IC_50_ value was derived from the fitted LogIC_50_ parameter as IC_50_ = $10^{\log{IC}_{50}}$. All data points from at least three independent experiments were included in the nonlinear regression analysis.

DPPH Assay: A 0.5 mL aliquot of sample solution was mixed with 4.0 mL of 0.1 mmol/L DPPH in ethanol. The mixture was kept in the dark at room temperature for 30 min, and the absorbance was measured at 517 nm. A control was prepared by mixing 0.5 mL ethanol with 4.0 mL DPPH solution. All experiments were performed in triplicate [34].

ABTS Assay: The ABTS radical cation was generated by reacting 7.4 mmol/L ABTS stock solution with 2.6 mmol/L potassium persulfate for 12 h in the dark. The resulting solution was then diluted with ethanol to an absorbance of 0.70 ± 0.02 at 734 nm. A 0.8 mL aliquot of the sample was mixed with 3.2 mL of the diluted ABTS^+^• solution. After 6 min, the absorbance was measured at 734 nm. Trolox (0.1 mg/mL) was used as a positive control. All experiments were performed in triplicate [35].

FRAP Assay: A 1.0 mL aliquot of the sample was mixed with 0.5 mL of phosphate buffer (100 mmol/L, pH 6.6) and 1.0 mL of 1% potassium ferricyanide. The mixture was incubated at 50 °C in a water bath (Shanghai Yuejin Medical Instrument Co., Ltd., Shanghai, China) for 30 min. Subsequently, 1.0 mL of 10% trichloroacetic acid was added, and the mixture was centrifuged at 1000 rpm for 8 min in a low-speed centrifuge. Finally, 2.0 mL of the supernatant was mixed with 0.28 mL of 0.5% FeCl_3_ and 1.0 mL of distilled water. After 10 min, the absorbance was measured at 700 nm. Higher absorbance values indicate greater reducing power. All experiments were performed in triplicate [36].

#### 2.5.2. Effects on TNF-α-Induced HFLS-RA Cells

The anti-RA activity evaluation was performed by integrating two sets of indicators measured in parallel from the same batch of treated cells: (1) inflammatory cytokine levels (IL-6, IL-8, IL-1β) detected by ELISA, reflecting the anti-inflammatory effect; (2) cellular metabolic viability detected by CCK-8 assay, reflecting synoviocyte activation status and providing a reference for excluding non-specific cytotoxic interference.

Stock solutions of formononetin were prepared in dimethyl sulfoxide (DMSO) and stored at −20 °C. Before treatment, stock solutions were diluted in complete culture medium to the indicated final concentrations. The final DMSO concentration in all treatment groups was ≤0.1% (*v*/*v*), and a solvent control group containing 0.1% DMSO in complete medium was included in all assays.

HFLS-RA cells were cultured following the method described by R. Ian Freshney [37]. The HFLS-RA cell line was obtained from Huatuo Bio-Technology Co., Ltd., Guangzhou, China, and authenticated by STR profiling and synovial cell marker (vimentin, fibronectin) identification by the supplier. Cells from passages 4–7 were used for all experiments, as this passage range maintains stable morphology and inflammatory responsiveness according to our pre-experiments and published protocols. Cells were cultured in DMEM-H medium supplemented with 10% fetal bovine serum (FBS) and 1% penicillin–streptomycin at 37 °C in a humidified atmosphere containing 5% CO_2_. Cells were passaged using 0.25% trypsin-EDTA and cryopreserved in a freezing medium consisting of 50% basal medium, 40% FBS, and 10% DMSO.

For the CCK-8 assay, cells were seeded in 96-well plates at a density of 2 × 10^3^ cells per well and cultured overnight. Cells were treated with different concentrations (0, 1, 2.5, 10, 50, 100, 250, 500, 1000 µg/mL) of formononetin for 24, 48, and 72 h. Independent blank control groups, solvent control groups, and TNF-α model control groups were set up in parallel at each time point, and all sample groups were compared with the model control at the corresponding time point. The cell viability of untreated blank controls was defined as 100% at each time point, and cells maintained normal growth throughout the 72 h culture period. After treatment, the medium was replaced with 100 µL of fresh medium containing 10 µL of CCK-8 reagent. The plates were incubated for 2 h, and the absorbance was measured at 450 nm using a microplate reader (Varioskan LUX, Thermo Scientific, Waltham, MA, USA). Cell viability was calculated relative to the blank control group. It should be noted that the CCK-8 assay measures cellular metabolic activity rather than absolute cell number; thus, the results reflect cell viability status and cannot be directly equated to proliferation inhibition. Each experiment was performed in triplicate. HFLS-RA cells in the logarithmic growth phase were seeded into 6-well plates at a density of 5 × 10^4^ cells/mL by adding 200 μL of cell suspension to each well. Subsequently, 20 ng/mL TNF-α was added, and the cells were incubated at 37 °C with 5% CO_2_ for 2 h. A 2 h pre-stimulation period was selected to allow cells to enter an inflammatory state prior to drug intervention, which better mimics the pathological process of synovial inflammation in RA. The TNF-α concentration of 20 ng/mL was chosen based on classic published protocols for the HFLS-RA inflammatory model and verified by our pre-experiments to stably induce high expression of pro-inflammatory cytokines and abnormal synoviocyte activation. Following incubation, samples or drugs at the designated concentrations were added according to the experimental design. The experimental groups were set up as follows: a blank control group (HFLS-RA cells in complete medium), a TNF-α model group (treated with 20 ng/mL TNF-α), a positive drug control group (50 μmol/L hydroxychloroquine + 20 ng/mL TNF-α; this concentration was selected based on published in vitro studies using HFLS-RA cells and is used for model validity verification only, not for direct clinical potency comparison), and sample intervention groups. The sample intervention groups received formononetin at three dose levels: high (150 μg/mL), medium (100 μg/mL), and low (50 μg/mL), each added after TNF-α induction.

After incubation for 24, 48, and 72 h, the cell culture supernatants were collected. The levels of inflammatory cytokines (IL-6, IL-8, and IL-1β) were measured using specific ELISA kits according to the manufacturer’s instructions. The absorbance was read at 450 nm using the microplate reader. All experiments were independently repeated three times.

The effect of the flavonoid on the proliferation of TNF-α-induced HFLS-RA cells was investigated using the CCK-8 assay. The cell proliferation rate was calculated, and all experiments were performed in triplicate.

### 2.6. Statistical Analysis

Experimental design and statistical analyses were performed using Design-Expert 13 Trial, SPSS 26.0, OriginPro 2019b, Excel 2021, and R 4.4.3 software. All data were subjected to Shapiro–Wilk normality test and Levene’s homoscedasticity test prior to parametric analysis. One-way ANOVA followed by Duncan’s multiple range test was adopted for intergroup comparisons. All experiments included 3 independent biological replicates. Data are presented as mean ± standard deviation, with *p* < 0.05 considered statistically significant.

## 3. Results and Discussion

### 3.1. Ethanol Reflux Extraction and Optimization of Crude Extract for Flavonoid Enrichment

#### 3.1.1. Single-Factor Experiments on Ethanol Reflux Extraction

**Figure 1:**
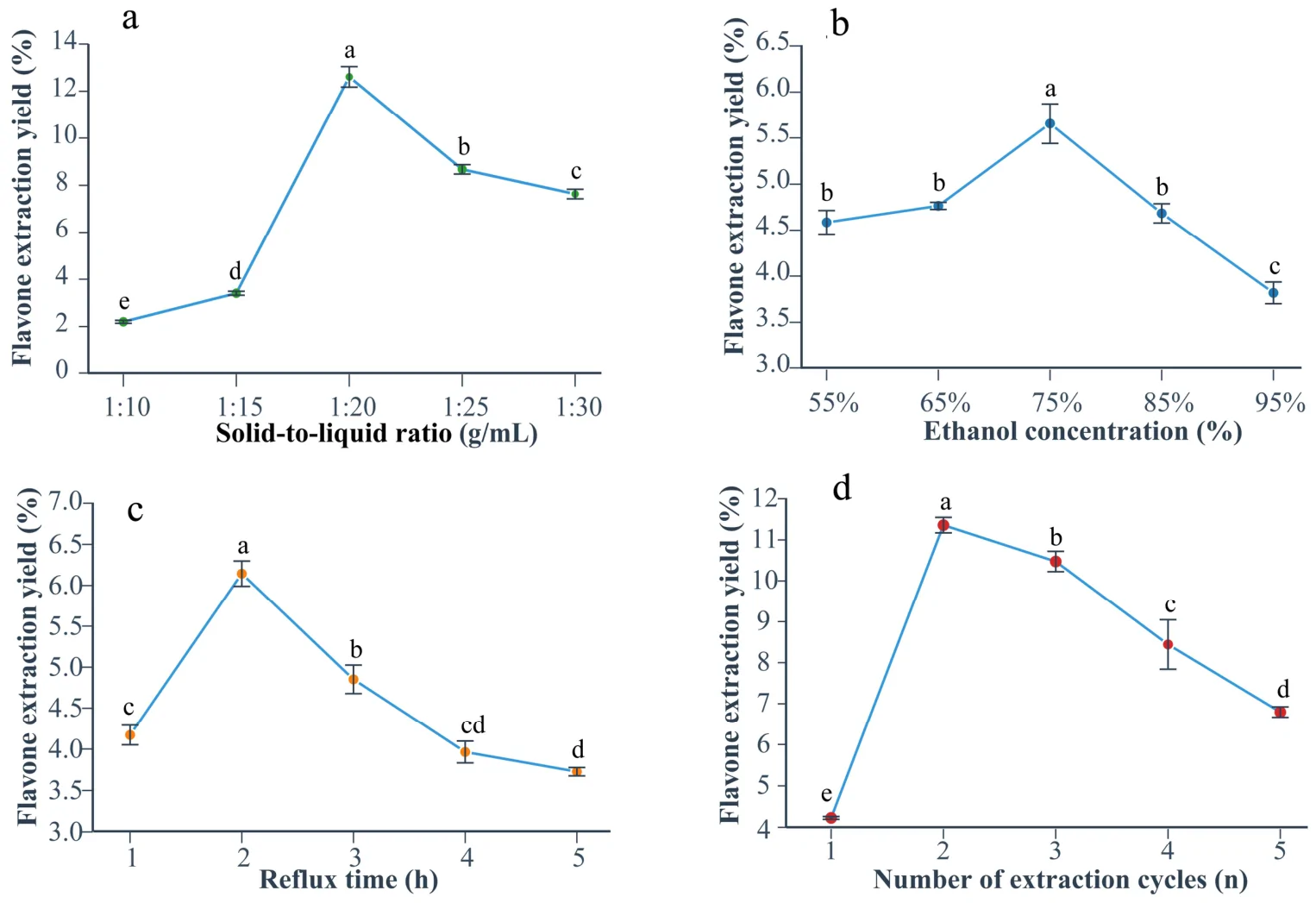
Effects of single factors on flavonoid extraction yield from *Lycopus lucidus* Turcz. var. *hirtus* Regel. (**a**) Solid-to-liquid ratio (g/mL); fixed baseline conditions: ethanol concentration 75%, reflux time 1 h, extraction cycles = 1. (**b**) Ethanol concentration (%); fixed baseline conditions: solid-to-liquid ratio 1:20 g/mL, reflux time 1 h, extraction cycles = 1. (**c**) Reflux time (h); fixed baseline conditions: solid-to-liquid ratio 1:20 g/mL, ethanol concentration 75%, extraction cycles = 1. (**d**) Number of extraction cycles (*n*); fixed baseline conditions: solid-to-liquid ratio 1:20 g/mL, ethanol concentration 75%, reflux time 1 h. Data are presented as mean ± SD (*n* = 3). Different lowercase letters above bars indicate statistically significant differences at *p* < 0.05 by one-way ANOVA followed by Duncan’s multiple range test. The y-axis represents flavonoid yield of each individual extraction cycle not cumulative total yield (%).

This study employed the ethanol reflux method to obtain a crude extract from *L. lucidus* var. *hirtus*. Four parameters—solid-to-liquid ratio, ethanol concentration, reflux time, and number of extraction cycles—were optimized through single-factor experiments. The variations in extraction yield are illustrated in Figure 1.

The solid-to-liquid ratio is a key factor affecting flavonoid extraction yield [38]. As shown in Figure 1a, the extraction yield increased to 12.4% at a ratio of 1:20 and then declined with further increases. Insufficient solvent limits flavonoid dissolution, while an excessively high ratio co-extracts more hydrophilic impurities and may accelerate flavonoid degradation [39,40,41,42]. Accordingly, a solid-to-liquid ratio of 1:20 was selected for subsequent experiments.

Ethanol concentration modulates solvent polarity and thus the solubility of target flavonoids [43]. As shown in Figure 1b, the extraction yield rose from 4.6% to 5.7% as ethanol concentration increased from 55% to 75%, then decreased at higher concentrations. Moderate ethanol levels promote solvent penetration into plant cell walls; excessively high concentrations may cause cell dehydration and protein denaturation, hindering flavonoid diffusion [44,45]. The optimal ethanol concentration was determined as 75%.

Reflux time markedly affects mass transfer efficiency and extraction yield, and follows a rise-then-fall trend [46,47]. As shown in Figure 1c, the yield increased from 4.2% to 6.2% when reflux time was extended from 1 to 2 h, then decreased with further prolongation. The initial increase is driven by solvent penetration and a steep concentration gradient, while the subsequent decline results from thermal degradation of flavonoids and increased dissolution of hydrophilic impurities under prolonged heating [41,43,48,49]. The optimal reflux duration was set at 2 h.

Increasing extraction cycles extends solid-solvent contact time and facilitates flavonoid diffusion from cells [50]. As shown in Figure 1d, the yield increased from 4.1% to 11.3% when cycles rose from 1 to 2, then decreased with additional cycles. The improvement with the second cycle benefits from fresh solvent and a renewed concentration gradient; further cycles lead to flavonoid oxidation, thermal degradation, and complex formation with macromolecular impurities [51,52,53,54]. Thus, 2 extraction cycles were selected as the optimum.

#### 3.1.2. Response Surface Methodology Results for Extraction of Crude Extract for Flavonoid Enrichment from *Lycopus lucidus* Turcz. var. *hirtus* Regel by Ethanol Heating Reflux

Based on the single-factor experimental results, RSM was employed to optimize the ethanol reflux extraction process of crude extract, aiming to maximize the yield of rutin-equivalent phenolic components enriched in flavonoids. A quadratic equation model was obtained through nonlinear regression fitting (Table 2): Y(%) = 8.91 + 0.28A + 0.221B − 0.046C − 0.011D − 0.339AB − 0.528AC + 0.093AD − 0.345BC + 0.039BD + 0.135CD − 3.04A^2^− 4.76B^2^− 2.04C^2^− 1.17D^2^.

As shown in Table 3, a four-factor, three-level response surface design was implemented with the solid-to-liquid ratio (A), ethanol concentration (B), reflux time (C), and number of reflux cycles (D) as the independent variables, in order to investigate the interactions AB, AC, AD, BC, BD, and CD. Analysis of the model’s significance revealed that, for the extraction yield of total flavonoids, the solid-to-liquid ratio (A) had a highly significant effect (*p* < 0.01), ethanol concentration (B) had a significant effect (*p* < 0.05), and the quadratic terms of all four factors (A^2^, B^2^, C^2^, D^2^) exhibited highly significant effects on the yield (*p* < 0.01). Regarding factor interactions, the AB and BC interactions were significant (*p* < 0.05), while the AC interaction was highly significant (*p*< 0.01). Based on the F-values presented in the table, the order of influence of the factors was: ethanol concentration (B) > solid-to-liquid ratio (A) > reflux time (C) > number of reflux cycles (D). The overall model was highly significant (*p* < 0.01) with a non-significant lack-of-fit term (*p* > 0.05), indicating good fitting performance and reliable predictive ability.

**Figure 2:**
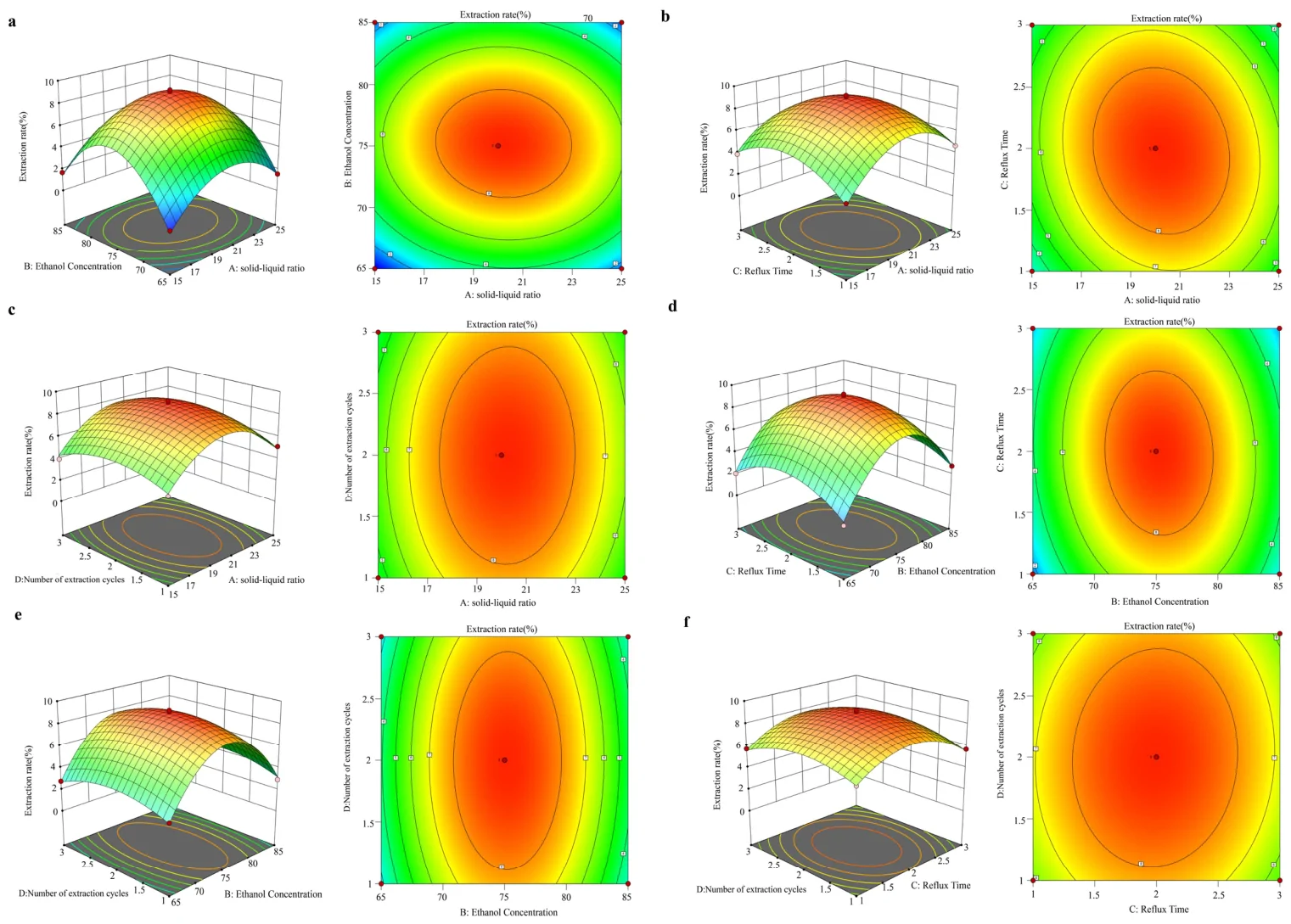
Interaction effects and contour plots for flavonoid yield of *Lycopus lucidus* Turcz. var. *hirtus* Regel extract. (**a**) Solid-to-liquid ratio and ethanol concentration; (**b**) Solid-to-liquid ratio and reflux time; (**c**) Solid-to-liquid ratio and number of extraction cycles; (**d**) Ethanol concentration and reflux time; (**e**) Ethanol concentration and number of extraction cycles; (**f**) Reflux time and number of extraction cycles.

The interaction effects between factors were further analyzed by response surface and contour plots. Figure 2a,d showed markedly elliptical contours, corresponding to significant (*p* < 0.05) or highly significant (*p* < 0.01) interactions; Figure 2c,e,f presented nearly circular contours, indicating non-significant interactions between the corresponding factor pairs.

As shown in Figure 2a, the interaction between solid-to-liquid ratio and ethanol concentration was significant. The extraction yield increased prominently with the rise in both factors within a certain range, but decreased sharply when the thresholds were exceeded. For the interaction between solid-to-liquid ratio and reflux cycles (Figure 2c), the yield increased gently with both factors and declined after 2 cycles, with no significant interaction observed. Excessive cycles prolong heat exposure and may cause flavonoid degradation, thus reducing the apparent yield [55,56].

Figure 2b showed a highly significant interaction between solid-to-liquid ratio and reflux time. When reflux time was below 2 h, increasing both factors sharply elevated the extraction yield; when reflux time exceeded 2 h, the yield decreased markedly. An appropriate solid-to-liquid ratio provides sufficient dissolution driving force, while moderate reflux time ensures complete flavonoid release without obvious thermal degradation, thus exerting a combined promotion effect [57]. The optimal combination of solid-to-liquid ratio and reflux time derived from the model was 1:20.38 and 2 h, respectively. The optimal extraction parameters solved by Design-Expert 13 software were as follows: solid-to-liquid ratio 1:20.38, ethanol concentration 75.25%, reflux time 2.0 h, and 2 reflux cycles. For practical operation, the parameters were adjusted to solid-to-liquid ratio of 1:20, ethanol concentration of 75%, reflux time of 2.0 h, and 2 cycles. Three verification experiments were performed under these conditions, giving an average extraction yield of 9.1 ± 0.83% (rutin equivalents), with a relative error of only 2.1% compared with the predicted value, confirming the reliability of the model. Compared with the RSM-optimized ethanol reflux method reported by Li et al. (yield 9.08 ± 0.91%), the present process achieved a comparable or slightly higher extraction efficiency [23].

### 3.2. Purification of Flavonoids from Lycopus lucidus Turcz. var. hirtus Regel

#### 3.2.1. Selection of the Optimal Macroporous Resin

Macroporous resins are widely used for natural product purification due to their good selectivity, easy elution, reusability, and low cost [58,59,60,61]. They are classified by polarity [62], and their porous structure enables size- and polarity-dependent retention [63] through physical and chemical interactions [64]. Therefore, this study selected five macroporous resins with different polarities—NKA-9, AB-8, H103, D4020, and D101—and screened the optimal resin by comparing their adsorption and desorption rates.

**Figure 3:**
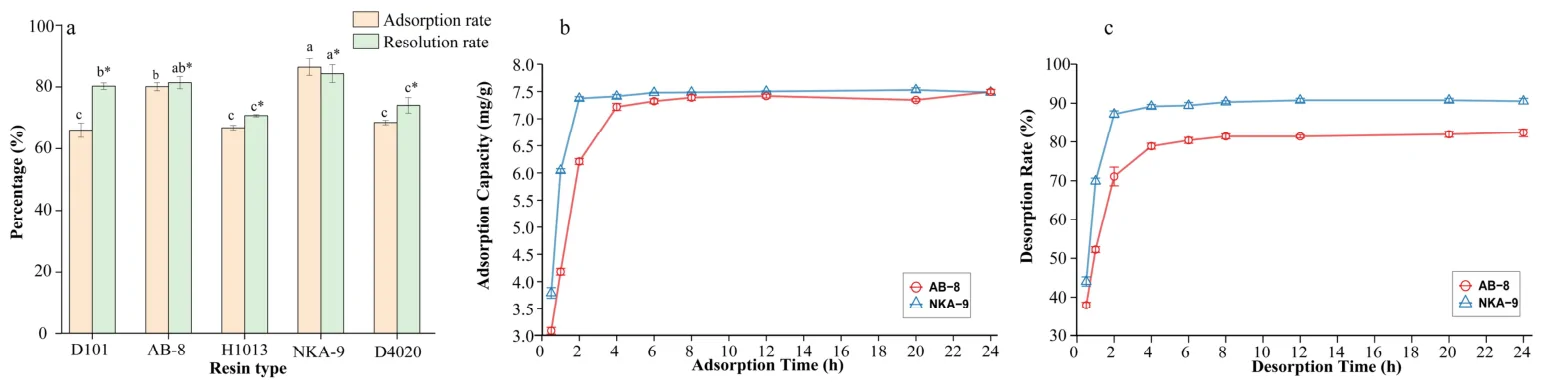
Screening and evaluation of adsorption performance of macroporous resins for total flavonoids from *Lycopus lucidus* Turcz. var. *hirtus* Regel. (**a**) Comparison of static adsorption–desorption performance of five resins; (**b**) Static adsorption kinetics curves of NKA-9 and AB-8 resins; (**c**) Dynamic adsorption breakthrough curves. Different lowercase letters above bars indicate significant differences among groups by one-way ANOVA followed by multiple comparisons (*p* < 0.05), and asterisks (*) denote significant differences between the two indicators within the same group (*p* < 0.05).

As shown in Figure 3a, under identical treatment conditions, the order of adsorption rates for the five resins was NKA-9 > AB-8 > H103 > D4020 > D101. The adsorption rates of AB-8 and NKA-9 resins were 80.095 ± 1.282% and 86.374 ± 2.691%, respectively, both exceeding 80%, while their desorption rates were 81.382 ± 1.991% and 84.280 ± 2.854%, respectively. The adsorption and desorption rates of AB-8 and NKA-9 resins for flavonoids were significantly higher than those of the other resins. Therefore, these two resins were selected as candidates for further screening. Resins with larger specific surface areas and pore diameters tend to exhibit higher adsorption performance [65]. Among them, AB-8 macroporous adsorption resin is a weakly polar resin known for its good adsorption properties [66], while NKA-9 resin is a typical polar adsorption resin that demonstrates unique advantages in separating polar natural products [67]. AB-8 (130–140 nm) and NKA-9 (155–165 nm) possess the largest average pore diameters, and their higher desorption rates can be attributed to the fact that larger pore sizes facilitate the desorption of flavonoids [68].

As shown in Figure 3b,c, the adsorption and desorption kinetics of the two resins were compared. NKA-9 resin reached adsorption equilibrium relatively early. At 2 h, its adsorption capacity reached 7.42 ± 0.03 mg/g, and further increases in time led to almost no change, with only a marginal difference from the maximum adsorption capacity of 7.57 ± 0.04 mg/g achieved at 20 h, which could be considered the adsorption plateau. Similarly, its desorption rate reached a plateau of 87.19 ± 0.84% at around 2 h. By contrast, AB-8 resin reached a plateau adsorption capacity of 7.26 ± 0.06 mg/g at 4 h, and its desorption rate plateaued at 78.86 ± 0.76% at 4 h. Compared with NKA-9 resin, AB-8 resin exhibited a lower adsorption capacity, a lower desorption rate, and longer times required to reach both adsorption and desorption equilibrium. Therefore, NKA-9 resin was considered more effective and was selected as the optimal macroporous adsorption resin for purifying total flavonoids from *L. lucidus* var. *hirtus*. This superior performance may be attributed to the larger pore size of NKA-9, which reduces the diffusion resistance of flavonoid molecules within the pores [69]. This finding is consistent with the results of Zhang et al. on bound polyphenols, who also concluded that NKA-9 performed better than AB-8 resin [70].

#### 3.2.2. Optimization of the Purification Process for Flavonoids from *Lycopus lucidus* Turcz. var. *hirtus* Regel

**Figure 4:**
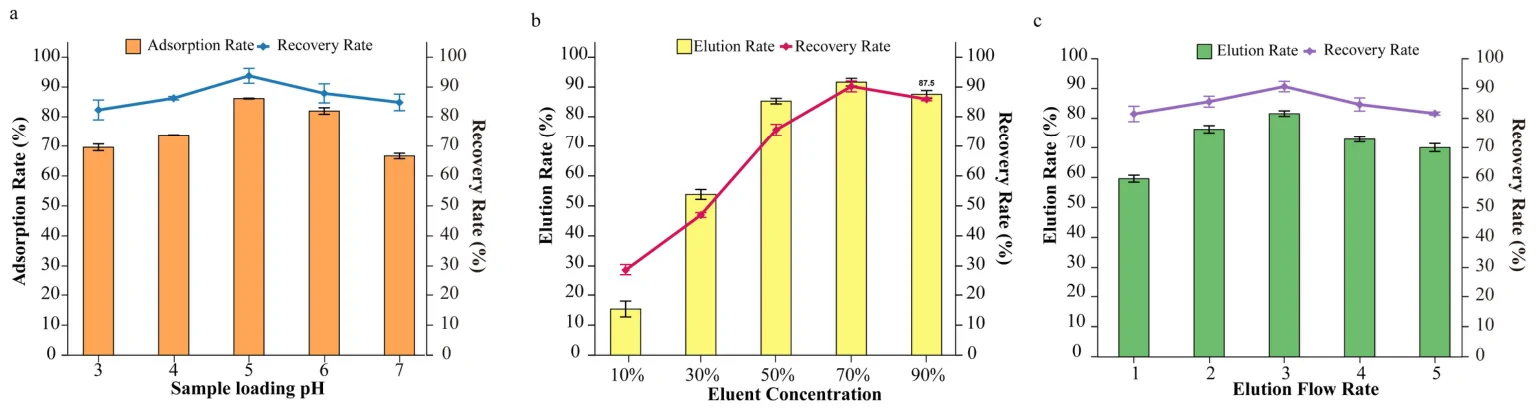
Single-Factor Tests for Macroporous Resin Purification of Flavonoids from *Lycopus lucidus* Turcz. var. *hirtus* Regel. (**a**) Sample loading pH; (**b**) Eluent concentration; (**c**) Elution flow rate.

Dynamic adsorption–desorption parameters of macroporous resins, such as the pH of the sample solution, sample loading flow rate, eluent concentration, and elution flow rate, can all affect the adsorption–desorption performance of the resin [71]. In this study, single-factor experiments were conducted to optimize the purification of flavonoids from *L. lucidus* var. *hirtus* using macroporous resin, focusing on three factors: sample pH, ethanol concentration in the eluent, and elution flow rate. The results are presented in Figure 4.

Figure 4a illustrates the effect of sample pH on the purification of flavonoids from *L. lucidus* var. *hirtus*. The adsorption rate was determined in adsorption experiments using sample solutions with different pH values, and the maximum adsorption rate was achieved at pH 5. The recovery rate was measured in separate desorption experiments conducted under optimized elution conditions, and it was also highest when the corresponding sample pH was 5. As the pH decreased or increased, both rates declined. As the pH decreased or increased, both rates began to decline. Therefore, a sample solution with a pH of 5 was found to be more favorable for adsorption by the macroporous resin, yielding an average adsorption rate of 85.92% and an average recovery rate of 93.78%. This is because flavonoid molecules contain multiple phenolic hydroxyl groups, whose degree of dissociation is directly influenced by the solution pH [72]. At a moderate pH, the protonation degree of the phenolic hydroxyl groups is higher, reducing the overall molecular polarity and allowing the hydrophobic skeleton to be more readily exposed. Under these conditions, the hydrophobic surface of the macroporous resin can form stable interactions with the hydrophobic regions of the flavonoid molecules, significantly enhancing adsorption efficiency [73]. Conversely, when the pH is too high, the phenolic hydroxyl groups tend to dissociate into phenolate ions under alkaline conditions, leading to electrostatic repulsion with the negatively charged resin surface and a reduction in adsorption efficiency [74]. Therefore, a pH of 5 was selected for subsequent experiments.

As shown in Figure 4b, when the ethanol concentration reached 70%, the recovery rate was maximized, with an average adsorption rate of 85.27% and an average recovery rate of 90.23%. Further increases in ethanol concentration resulted in a decrease in the adsorption rate. Low-concentration ethanol solutions have a weak ability to promote the adsorption of flavonoids onto the resin and are less effective in eluting flavonoids. According to the principle of “like dissolves like,” although high-concentration ethanol solutions have a polarity similar to that of flavonoids, they do not fully dissolve the flavonoids. Instead, they may elute more macromolecular impurities, thereby reducing purification efficiency [75]. Therefore, an ethanol concentration of 70% was chosen for subsequent experiments.

As shown in Figure 4c, when the elution flow rate reached 3 mL/min, the adsorption and recovery rates of the macroporous resin were the highest, with average values of 81.42% and 90.71%, respectively. Within the flow rate range of 1–3 mL/min, both rates gradually increased with increasing flow rate. However, within the range of 3–5 mL/min, both rates decreased as the flow rate increased. Increasing the elution flow rate tends to reduce the desorption rate gradually because a higher flow rate shortens the contact time between the eluent and the resin surface, preventing the full displacement of flavonoids adsorbed on the resin and leading to incomplete elution [76]. In contrast, a lower elution flow rate facilitates the desorption of flavonoids from the resin, thereby improving the desorption rate. However, an excessively low elution flow rate significantly prolongs the elution process, extending the overall production cycle and reducing efficiency [77]. Therefore, in practical applications, a balance must be struck between the desorption rate and production efficiency to determine the optimal elution flow rate [22]. In summary, an elution flow rate of 3 mL/min was selected for subsequent experiments.

The results of the orthogonal test for purifying flavonoids from *L. lucidus* var. *hirtus* using macroporous resin are presented in Table 4. Based on the range (R) analysis, the order of influence of the factors on the flavonoid recovery rate was C > B > A, i.e., elution flow rate > eluent concentration > sample pH. The results indicated that the optimal purification conditions were A_2_B_2_C_2_, corresponding to an elution flow rate of 3 mL/min, an eluent concentration of 70%, and a sample pH of 5, which yielded the highest purification efficiency. Three purification experiments were conducted under these optimized conditions. The average flavonoid recovery rate was 96.02 ± 0.92%, showing minimal difference from the model-predicted value of 96.21%. This close agreement confirms the reliability of the model.

Lyophilized samples of the crude flavonoid extract before column purification and the eluent after column purification, each of equal mass, were prepared. Under the premise of identical sample masses, the flavonoid content of both was determined in triplicate. The criterion for flavonoid purity was defined as the flavonoid content divided by the sample mass. The extraction rate and recovery rate were calculated for the pre- and post-column samples, respectively, and the effectiveness of the purification process was comprehensively evaluated based on these results. According to Table 5, after treatment with the optimized purification process, the average content of rutin-equivalent phenolic substances from *L. lucidus* var. *hirtus* increased from 44.64% before purification to 89.40% (both determined via aluminum-nitrite colorimetric assay), representing an approximately two-fold enrichment efficiency. Due to the non-specific cross-reactivity of this assay with phenolic acids and other polyphenols, flavonoid existence in var. cannot be concluded merely by colorimetric results. The definitive scientific evidence confirming the presence of flavonoid compounds in this under-investigated taxon is provided by our subsequent MPLC-HPLC isolation combined with comprehensive UV, IR, HR-MS, and NMR spectroscopic identification, which successfully identified formononetin, a typical isoflavone monomer. Therefore, this purification protocol effectively enriches flavonoid-type phenolic components of the herb. Notably, since the aluminum nitrate colorimetric method also responds to co-existing phenolic substances, the purity values reflect the relative enrichment degree of flavonoid-like components, rather than the absolute mass percentage of pure flavonoid compounds. For comparison, Ye Jiafeng et al. used HPD400 macroporous resin to purify total flavonoids from camellia husk and found that the purity increased from 19.62% to 39.16% [25]. Wang Jiaqi et al. employed AB-8 macroporous resin to purify flavonoids from *Polygonatum*, and the results showed that the purity increased to 5.31%, which was 11.8 times the concentration in the crude extract, along with a significant improvement in antioxidant activity [76]. It is evident that the purification efficacy of macroporous resins varies for different compounds from different biological sources. Therefore, optimizing the purification process for flavonoids from *L. lucidus* var. *hirtus* using macroporous resin is necessary.

### 3.3. Isolation and Identification of Flavonoid from Lycopus lucidus Turcz. var. hirtus Regel

#### 3.3.1. Determination of Flavonoid Ratios in *Lycopus lucidus* Turcz. var. *hirtus* Regel by HPLC

**Figure 5:**
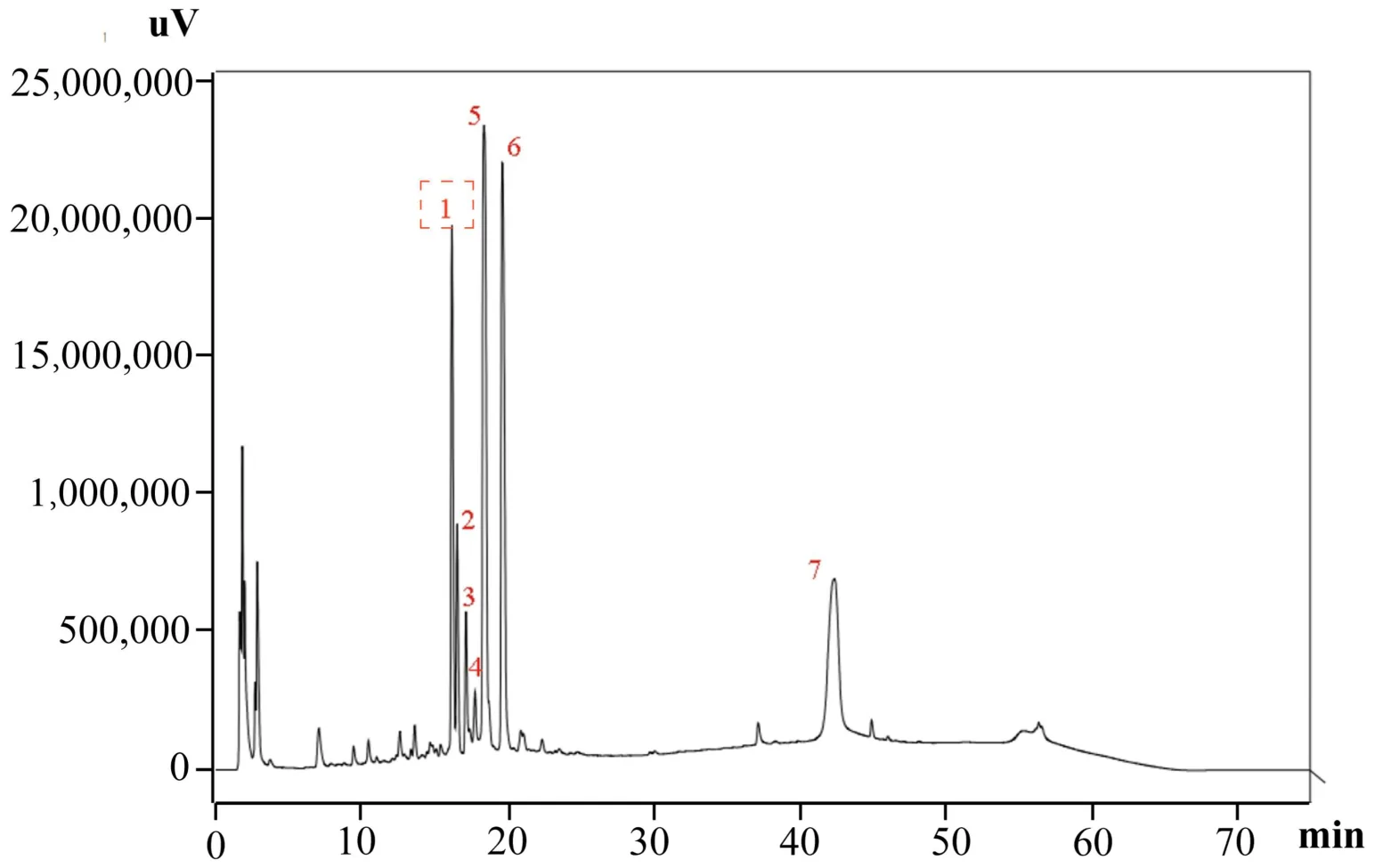
HPLC chromatogram of purified total flavonoids from *Lycopus lucidus* Turcz. var. *hirtus* Regel.

HPLC achieves efficient separation and qualitative/quantitative analysis based on solute distribution between stationary and mobile phases [78]. To clarify the chemical composition of phenolic constituents enriched in flavonoid components from *L. lucidus* var. *hirtus* and guide subsequent isolation and purification, HPLC analysis was first performed on the purified total flavonoid fraction after NKA-9 macroporous resin enrichment. As shown in Figure 5, the components in the total flavonoids eluted predominantly within the first 20 min. A solvent peak appeared at 2 min, and sample detection peaks began to emerge at 6 min. The more prominent peaks in the chromatogram were labeled as peaks 1 through 7. Peak 1 had a retention time of 16.314 min and a peak area of 31,117,492; Peak 2: 17.271 min, 11,027,036; Peak 3: 17.520 min, 5,540,145; Peak 4: 17.866 min, 11,362,657; Peak 5: 18.549 min, 43,359,618; Peak 6: 19.769 min, 42,682,832; Peak 7: 37.240 min, 8,063,155. Based on the peak area ratios, the relative peak area proportion of these seven peaks was approximately 6:1:1:2:8:8:1. This ratio is derived from peak area normalization at 360 nm and does not represent the true mass ratio, due to differences in molar extinction coefficients among different components.

#### 3.3.2. Isolation and Identification Results of Flavonoids from *Lycopus lucidus* Turcz. var. *hirtus* Regel

**Figure 6:**
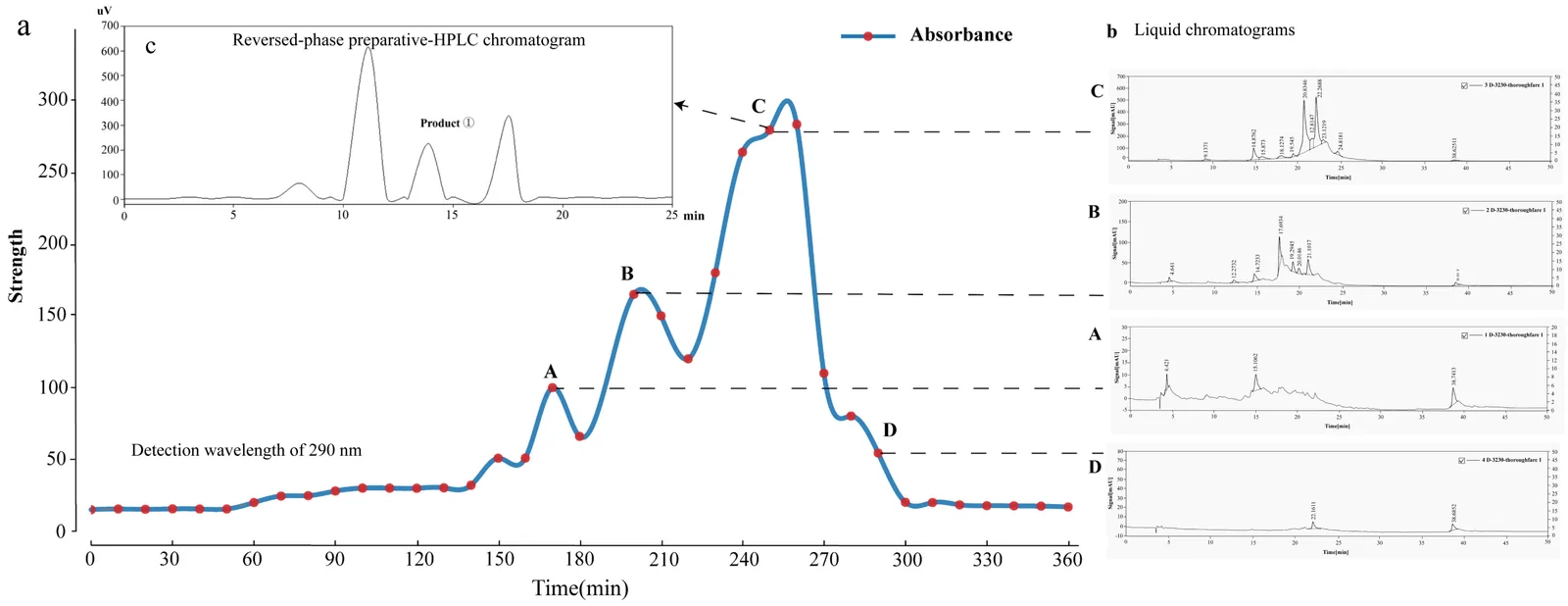
Liquid chromatographic analysis of fractionated components from *Lycopus lucidus* Turcz. var. *hirtus* Regel flavonoids. (**a**) Separation of *Lycopus lucidus* Turcz. var. *hirtus* Regel flavonoids using an LH-20 gel chromatography column; (**b**) Liquid chromatograms of Fractions A–D; (**c**) Reversed-phase HPLC analysis of Fraction C.

Based on the HPLC analysis results from Section 3.3.1, which indicated that the main active components of *L. lucidus* var. *hirtus* flavonoids were concentrated in the chromatographic region before 20 min, it was hypothesized that effective elution might be achieved using a methanol concentration around 20%. Therefore, an LH-20 gel chromatography column was first employed for intermediate separation. The results of the gradient elution are shown in Figure 6a. Fractions 1–18 collected between 0 and 180 min were pooled as Component A, with tube 17 showing the strongest signal, indicating the concentration of major constituents in this component. Fractions 19–22 collected between 180 and 220 min were labeled as Component B. A distinct peak with the strongest signal appeared between 220 and 270 min, corresponding to fractions 23–27, which were labeled as Component C and hypothesized to contain the main flavonoid components of *L. lucidus* var. *hirtus*. The irregular baseline between 270 and 300 min suggested the possible elution of other flavonoid compounds; this portion was collected as Component D. It was inferred that the main flavonoid components were present in Component C, although specific constituents required further analysis.

As shown in Figure 6b, Components A and D contained the least amount of flavonoids, with signal intensities for all peaks in these two components not exceeding 20 mAU. Component B contained more flavonoids compared with Components A and D, but the signal intensity of its highest peak was also below 150 mAU. In contrast, the peaks in Component C were sharp and distinct, with the highest peak signal intensity exceeding 600 mAU, indicating that the primary constituents of *L. lucidus* var. *hirtus* flavonoids were present in Component C. Therefore, further separation was focused on Component C.

HPLC analysis of each component (Figure 6c) revealed that the signal intensity of Component C was significantly higher than that of the other components (highest peak > 600 mAU), and its peak elution times corresponded to the major peak cluster before 20 min in the total flavonoid chromatogram. This confirmed that the main components of *L. lucidus* var. *hirtus* flavonoids were enriched in Component C. High-performance liquid chromatography was used for further separation of Component C. Given that preliminary gradient elution showed the main components eluted at approximately 20% methanol, 20% methanol was selected as the mobile phase for isocratic elution to optimize separation. Under these conditions, four sub-fractions were successfully separated. The chromatographic peak at 13.87 min was sharp and symmetrical, suggesting it was a high-purity monomer. Consequently, the eluate from 13 to 14 min was collected and named Isolated Product ① for subsequent structural identification and bioactivity studies.

Isolated Product ① was identified as formononetin by combined UV, IR, LC-MS, and NMR analyses. UV absorptions at 249 and 302 nm indicated an isoflavone skeleton, and IR bands confirmed hydroxyl (3433.99 cm^−1^) and carbonyl (1734.43 cm^−1^) groups. Negative-ion ESI-MS gave [M−H]^−^ at *m/z* 265.0952, consistent with the molecular formula C_16_H_12_O_4_, while ^1^H and ^13^C NMR data revealed characteristic methoxy signals (δH 3.77; δC 55.16) and an isoflavone carbonyl carbon at δC 174.65. All spectroscopic data matched those reported for formononetin [79,80].

### 3.4. Antioxidant Activity of Formononetin from Lycopus lucidus Turcz. var. hirtus Regel and Its Effect on TNF-α-Induced HFLS-RA Cells

#### 3.4.1. Determination of Antioxidant Activity

**Figure 7:**
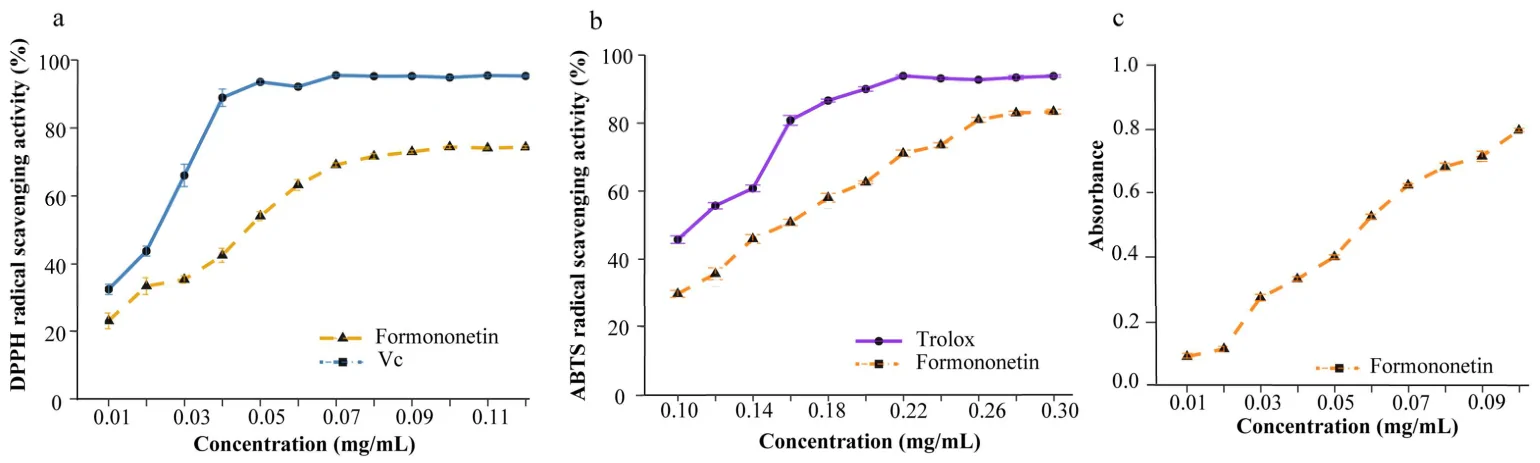
Antioxidant capacity determination of formononetin. (**a**) DPPH radical scavenging rate; (**b**) ABTS radical scavenging rate; (**c**) Ferric reducing antioxidant power (FRAP). Vc: ascorbic acid.

Antioxidant activity is one of the important characteristics of natural products [81]. DPPH scavenging capacity is a commonly used method for evaluating the antioxidant properties of herbal extracts [82]. The DPPH radical scavenging rates of formononetin—the flavonoid isolated from *L. lucidus* var. *hirtus*—and the positive control ascorbic acid (Vc) are shown in Figure 7a. Within the tested concentration range, both Vc and formononetin exhibited dose-dependent scavenging effects on DPPH radicals. Vc presented a steeper dose–response trend: its scavenging rate rose rapidly at low concentrations and reached a plateau of over 90% at approximately 0.07 mg/mL. In comparison, formononetin showed a relatively moderate growth rate, with its scavenging rate gradually leveling off at around 75% when the concentration reached 0.10 mg/mL. The half maximal inhibitory concentration (IC_50_) of formononetin for DPPH radical scavenging was calculated as 0.047 mg/mL, which was higher than that of Vc (0.023 mg/mL), indicating that the DPPH scavenging capacity of formononetin is inferior to that of Vc. This result is consistent with the findings of Chen et al. [83], jointly confirming that formononetin possesses definite DPPH radical scavenging ability and exerts corresponding antioxidant activity.

The ABTS assay is widely used to evaluate total antioxidant capacity by reflecting electron transfer in both hydrophilic and lipophilic systems [84,85]. As illustrated in Figure 7b, the ABTS radical scavenging rates of Trolox (positive control) and formononetin both increased continuously with rising sample concentrations, showing a significant dose-effect relationship. Trolox maintained higher scavenging efficiency across the entire concentration gradient, and its scavenging rate exceeded 90% and entered a plateau phase at 0.20 mg/mL. The IC_50_ value of formononetin for ABTS radical scavenging was 0.158 mg/mL, slightly higher than that of Trolox (0.109 mg/mL), suggesting that formononetin has moderate ABTS radical scavenging activity. This conclusion is also supported by previously reported research results [86].

The reducing power assay reflects electron/hydrogen donation, with absorbance positively correlated with reducing capacity and antioxidant activity [87]. The reducing power curve of formononetin is presented in Figure 7c. Within the concentration range of 0.01 to 0.09 mg/mL, the absorbance value increased steadily with the elevation of formononetin concentration, showing a favorable linear dose-dependent trend. This result demonstrates that formononetin has a certain reducing capacity, which provides supplementary evidence for its antioxidant effect from another perspective. The antioxidant activity of formononetin is mainly derived from its typical isoflavone core structure: the phenolic hydroxyl group in its molecular structure can directly scavenge free radicals through hydrogen atom or electron donation, and it can also exert indirect antioxidant effects by regulating the expression and activity of endogenous antioxidant enzyme systems [88]. However, the lack of a Trolox positive control brings two main methodological limitations to these FRAP results. First, without this calibrated reference, Trolox equivalent antioxidant capacity (TEAC) cannot be derived, so the absolute reducing potency of formononetin cannot be quantified and cross-study comparability of the data is limited. Second, potential systematic bias in this single batch of experiments cannot be independently verified, which lowers the absolute reliability of raw absorbance values.

#### 3.4.2. Effects of Formononetin Isolated from *Lycopus lucidus* Turcz. var. *hirtus* Regel on TNF-α-Induced HFLS-RA Cells

Synovial hyperplasia in RA arises from abnormal activation of fibroblast-like synoviocytes (FLS) [89], which exhibit tumor-like proliferation, apoptosis resistance, migration/invasion, and pro-inflammatory secretion, promoting pannus formation [90]. Regulating HFLS-RA proliferation is thus a key therapeutic strategy. In this study, the effects of three samples on HFLS-RA cell viability were evaluated by CCK-8 assay. The concentration that yielded an inhibition rate of approximately 80% was selected as the optimal dose, and the adjacent concentrations were designated as low and high doses, respectively.

**Figure 8:**
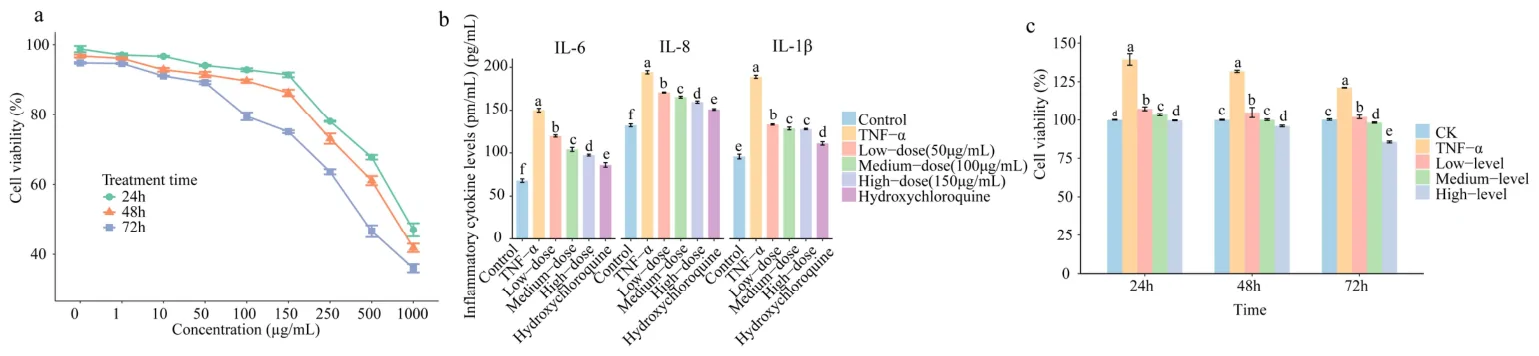
Effects of formononetin on cell viability and inflammatory phenotype of HFLS-RA cells. (**a**) Effects of different samples on the viability of normal HFLS-RA cells; (**b**) Effects of different samples on inflammatory cytokine levels in TNF-α-induced HFLS-RA cells; (**c**) Effects of different samples on the viability of TNF-α-induced HFLS-RA cells. Positive control: hydroxychloroquine (50 μmol/L). Different lowercase letters above bars indicate significant differences among groups by one-way ANOVA followed by multiple comparisons (*p* < 0.05).

As shown in Figure 8a, formononetin reduced HFLS-RA cell viability in both concentration- and time-dependent manners. Within the low concentration range of 0–100 μg/mL, cell viability remained above 90% across all three time points, indicating no obvious cytotoxicity in this interval. When the concentration exceeded 100 μg/mL, cell viability decreased sharply in a dose-dependent fashion and dropped below 50% at the highest concentration of 1000 μg/mL. Meanwhile, for any given treatment concentration, cell viability declined further as incubation time extended from 24 h to 72 h, confirming a notable time-dependent trend. Consistent with previous reports, formononetin has been demonstrated to exert anti-proliferative and anti-inflammatory effects on RA-FLS within a well-tolerated concentration range [91].

The inflammatory response in RA FLS is driven by excessive pro-inflammatory cytokines, including TNF-α, IL-6, IL-8, and IL-1β [92]. IL-6 promotes MMP-3 secretion and cartilage degradation [93], while IL-1β induces iNOS/NO production, leading to synovial hyperplasia and irreversible cartilage damage [94].

As shown in Figure 8b, TNF-α stimulation significantly elevated the secretion levels of IL-6, IL-8 and IL-1β in HFLS-RA cells compared with the control group, verifying successful establishment of the inflammatory injury model. All concentrations of formononetin exerted inhibitory effects on these inflammatory factors, with potency strengthening as dose increased, while the positive control hydroxychloroquine showed the strongest overall inhibitory effect among all groups. Specifically, formononetin treatment groups presented a clear dose-dependent inhibition pattern for all three detected cytokines. For IL-6 and IL-8, secretion levels decreased continuously from the low dose to the medium and high doses, showing a steady dose–response relationship. For IL-1β, the inhibitory effects of formononetin became relatively stable at medium and high doses, with no significant difference between the two dose groups, suggesting that the suppressive effect of formononetin on IL-1β secretion may approach a plateau at the medium dose level. Hydroxychloroquine, as a first-line clinical agent for rheumatoid arthritis, can significantly suppress the levels of multiple inflammatory factors, which was further confirmed in this study [95].

As shown in Figure 8c, the cell proliferation rate in the blank control group remained relatively stable around 100% throughout the 72 h observation period. In contrast, TNF-α stimulation maintained a markedly high level of cell proliferative activity at all three time points, indicating that TNF-α successfully induced sustained abnormal activation and proliferation of HFLS-RA cells. The three formononetin treatment groups (low, medium and high-dose) all reduced the proliferation rate of TNF-α-induced HFLS-RA cells compared with the TNF-α model group. This inhibitory effect intensified with increasing drug concentration and prolonged treatment time. Specifically, at 24 h, the proliferation rate in formononetin treatment groups was already significantly lower than that in the TNF-α group, indicating that formononetin could exert early regulatory effects on cell proliferative activity. At 48 h, the reduction in proliferation rate became more prominent than at 24 h, and differences among dose groups gradually became more distinguishable. At 72 h, the inhibitory effect on TNF-α-induced hyperproliferation was further enhanced, with the high-dose formononetin group exhibiting the strongest inhibitory activity among all treatment groups.

Collectively, these in vitro observations suggest that under the present experimental conditions, formononetin can suppress the abnormal proliferation of TNF-α-induced HFLS-RA cells without causing severe cytotoxicity. The time-dependent enhancement of the inhibitory effect may be related to multiple factors: gradual intracellular accumulation of formononetin to reach effective pharmacological concentrations, the time required for natural compounds to interfere with cell cycle progression and metabolic activity, as well as the persistent activation of inflammatory and proliferative signaling pathways in the TNF-α-induced cell model [96,97,98]. These findings are partially consistent with a previous study by Chen Jie et al., which reported anti-HFLS-RA activity of total flavonoids from *Lycopi Herba* [88], implying that formononetin may represent one of the potential active monomers contributing to the RA-relevant bioactivity of *Lycopi Herba* flavonoids.

### 3.5. Limitations of the Study

Several limitations of this study should be acknowledged. Regarding quantification methodology, total flavonoid content was determined by colorimetric assay on a rutin equivalent basis, which may overestimate the actual flavonoid mass due to cross-reactivity with co-occurring phenolic constituents. In this study, HPLC fingerprint analysis was implemented to qualitatively characterize flavonoid constituents and calculate relative peak-area ratios. Nevertheless, absolute quantitative HPLC analysis for each flavonoid was not performed. The experimental design of the present work mainly focused on the separation, purification, structural identification, and in vitro bioactivity evaluation of the target flavonoid. A complete set of HPLC quantitative method-validation experiments, including calibration curve establishment, precision, repeatability, stability, and spike-recovery tests, was not predefined within the experimental workflow. As a consequence, absolute mass contents of individual flavonoids in the total flavonoid fraction could not be acquired. Without these quantitative data, it is not feasible to precisely evaluate the real mass proportion of each constituent, which restricts accurate dissection of the contribution of each flavonoid to the bioactivity of the crude herbal extract. For cellular assays, the CCK-8 assay measures cellular metabolic activity rather than absolute proliferative capacity; cytokine levels were not normalized to total cellular protein content, and dedicated cytotoxicity assays (e.g., LDH release), definitive proliferation verification (e.g., EdU incorporation), and investigations into compound stability, solubility and cellular uptake in culture medium were not performed. Mechanistically, this study only documented inhibitory effects on pro-inflammatory cytokine production and cell viability, without interrogating classical RA-associated signaling pathways including NF-κB, MAPK, JAK-STAT and the NLRP3 inflammasome; such mechanistic studies are essential to understand the mode of action of formononetin. Additionally, no clinically used disease-modifying antirheumatic drugs (DMARDs) were included as positive controls to benchmark the magnitude of bioactivity.

Furthermore, the crude total flavonoid fraction was not tested in parallel in the HFLS-RA model, so the relative contribution of formononetin to the overall activity of the herbal extract cannot be quantitatively determined, and potential synergistic or antagonistic effects among multiple flavonoid components remain unexplored. Finally, all findings are derived exclusively from in vitro cell-based experiments. Given the generally low oral bioavailability and extensive in vivo metabolism of flavonoids, the therapeutic potential of formononetin must be further validated in animal models of RA and, ultimately, in clinical studies before any translational claims can be made. The in vitro antioxidant and anti-inflammatory effects observed in this study cannot be directly extrapolated to equivalent efficacy in vivo, as the complex physiological environment, metabolic transformation, and tissue distribution in living organisms may significantly alter the bioactivity and exposure level of the compound.

## 4. Conclusions

This study optimized the ethanol reflux extraction of the crude extract from *L lucidus* var. *hirtus* using response surface methodology and identified NKA-9 macroporous resin as an effective purification medium. From the purified fraction, formononetin was isolated as the major flavonoid, unequivocally characterized by multi-spectroscopic analysis, and its content was quantified in both the crude rhizome and the refined fraction. Functional assays demonstrated that formononetin exhibits moderate antioxidant activity and, within a non-cytotoxic concentration range, dose-dependently inhibits TNF-α-induced secretion of IL-6, IL-8, and IL-1β, as well as the aberrant metabolic activity of HFLS-RA cells. These in vitro results provide preliminary experimental evidence indicating that formononetin acts as an anti-inflammatory-active constituent of this ethnomedicinal herb. Such cellular observations cannot directly validate its clinical efficacy against rheumatoid arthritis, but offer supporting clues for its traditional ethnomedicinal application. The optimized extraction and purification protocols also offer a practical foundation for resource utilization. Future investigations are required to elucidate the underlying molecular pathways, evaluate its in vivo biological performance, assess pharmacokinetic properties, and evaluate potential synergistic effects among flavonoid components.

## Figures and Tables

**Table 1:** Physicochemical properties of macroporous resins.

Resin Type	Polarity	Specific Surface Area (m^2^/g)	Average Pore Diameter (A)
D101	Non-polar	520–550	25–28
NKA-9	Polar	250–290	155–165
D4020	Non-polar	540–580	100–105
AB-8	Weakly polar	480–520	130–140
H103	Non-polar	1000–1100	85–95

**Table 2:** Box–Behnken experimental design.

Box–Behnken Experimental Design and Results					
No.	A	B	C	D	Extraction Yield (%)
1	15	65	2	2	0.528
2	20	65	3	2	2.052
3	20	65	1	2	1.296
4	15	75	2	1	4.308
5	20	85	2	3	2.952
6	20	75	1	1	5.892
7	20	75	2	2	9.060
8	25	75	3	2	3.168
9	20	85	2	1	2.916
10	15	85	2	2	1.680
11	20	65	2	1	2.892
12	20	75	3	3	6.108
13	20	65	2	3	2.772
14	20	75	2	2	8.664
15	20	75	2	2	9.204
16	20	75	3	1	5.712
17	25	75	2	3	5.172
18	25	75	2	1	5.136
19	20	75	1	3	5.748
20	15	75	2	3	3.972
21	15	75	1	2	3.264
22	20	85	3	2	2.112
23	20	75	2	2	8.844
24	20	75	2	2	8.784
25	20	85	1	2	2.736
26	25	75	1	2	4.656
27	25	85	2	2	1.332
28	25	65	2	2	1.536
29	15	75	3	2	3.888

Note: A = solid-to-liquid ratio (g/mL); B = ethanol concentration (%); C = reflux time (h); D = number of extraction cycles.

**Table 3:** Analysis of variance for the regression model.

Analysis of Variance for the Regression Model						
Source	Sum of Squares	df	Mean Square	F-Value	*p*-Value	Significance
Model	186.2	14	13.3	151.98	<0.0001	***
A	1.39	1	1.39	15.83	0.0014	**
B	104.66	1	104.66	1195.92	<0.0001	***
C	0.11	1	0.11	1.25	0.2817	
D	0.01	1	0.01	0.07	0.7911	
AB	0.46	1	0.46	5.25	0.0379	*
AC	1.12	1	1.12	12.74	0.0031	**
AD	0.03	1	0.03	0.4	0.5396	
BC	0.48	1	0.48	5.44	0.0351	*
BD	0.01	1	0.01	0.07	0.7959	
CD	0.07	1	0.07	0.83	0.3768	
A2	59.75	1	59.75	682.79	<0.0001	***
B2	147.25	1	147.25	1682.65	<0.0001	***
C2	26.89	1	26.89	307.28	<0.0001	***
D2	8.84	1	8.84	101.05	<0.0001	***
Residual	1.23	14	0.09			
Lack of Fit	1.04	10	0.1	2.18	0.2349	
Pure Error	0.1897	4	0.0474			

Note: A = solid-to-liquid ratio (g/mL); B = ethanol concentration (%); C = reflux time (h); D = number of extraction cycles. * *p* < 0.05, ** *p* < 0.01, *** *p* < 0.001.

**Table 4:** Analysis of orthogonal experiment results for flavonoid purification from *Lycopus lucidus* Turcz. var. *hirtus* Regel.

No.	A: Sample pH	B: Eluent Concentration (%)	C: Elution Flow Rate (mL/min)	Recovery Rate (%)
1	3	3	1	75.95
2	1	2	3	86.24
3	3	1	3	76.85
4	1	3	2	84.28
5	2	3	3	82.59
6	3	2	2	91.61
7	2	2	1	91.86
8	2	1	2	95.66
9	1	1	1	70.9
k1	81.47	82.14	80.9	
k2	90.37	90.24	90.52	
k3	81.47	80.94	81.89	
R	8.9	9.3	9.61	

**Table 5:** Comparison of Purity and Yield of Total Flavonoids from *Lycopus lucidus* Turcz. var. *hirtus* Regel Purified by NKA-9 Macroporous Resin.

No.	Purified Eluate Purity (%)	Elution Recovery Rate (%)	Crude Extract Purity (Precolumn) (%)	Flavonoid Extraction Yield (%)
1	88.24	96.33	45.27	8.34
2	90.32	95.47	44.38	7.98
3	89.65	95.89	44.26	7.89
Mean	89.4	95.9	44.64	8.07
RSD (%)	1.19	0.45	1.24	2.95

## Data Availability

The data that support the findings of this study are available from the corresponding author upon reasonable request.

## Supplementary Materials

The following supporting information can be downloaded at: https://www.mdpi.com/article/10.3390/plants15172654/s1. Structural Identification of Flavonoid Monomers from *Lycopus lucidus* Turcz. var. *hirtus* Regel.

## Abbreviations

DMARDs	Disease-modifying antirheumatic drugs
DMSO	Dimethyl sulfoxide
ELISA	Enzyme-linked immunosorbent assay
FBS	Fetal bovine serum
FLS	Fibroblast-like synoviocytes
FRAP	Ferric reducing antioxidant power
HFLS-RA	Human rheumatoid arthritis fibroblast-like synoviocytes
HPLC	High-performance liquid chromatography
iNOS	Inducible nitric oxide synthase
*L. lucidus* var. *hirtus*	*Lycopus lucidus* Turcz. var. *hirtus* Regel
MPLC	Medium-pressure column chromatography
NO	Nitric oxide
NSAIDs	Non-steroidal anti-inflammatory drugs
PBS	Phosphate-buffered saline
RA	Rheumatoid arthritis
TNF-α	Tumor necrosis factor-alpha
