# Supplementary material for: Optimization of the Isolation and Purification of Formononetin from Lycopus lucidus Turcz. var. hirtus Regel, Structural Identification, and Its In Vitro Anti-Inhibitory Effects in TNF-α-Induced HFLS-RA Cells
Source: Plants (Basel). 2026 Aug 29;15(17):2654. doi: 10.3390/plants15172654 (PMC13566966; doi:10.3390/plants15172654)
Supplement: Supplementary file 1 [file plants-15-02654-s001.zip › plants-4479575-supplementary.pdf]

1. Structural Identification of Flavonoid Monomers from *Lycopus lucidus* Turcz. var. *hirtus* Regel

When deducing the structure of unknown compounds, techniques such as ultraviolet spectroscopy, infrared spectroscopy, mass spectrometry, and nuclear magnetic resonance spectroscopy are typically applied in combination. These methods collectively provide critical information and multiple lines of evidence for structural confirmation [80]. In this study, the structure of Isolated Product ① (Figure 7), obtained from the separation described in Section 3.3.2, was systematically identified using various analytical techniques, including UV, IR, LC-MS, and NMR. The compound was ultimately identified as formononetin, a typical isoflavone possessing a characteristic isoflavone backbone structure [81].

The UV spectrum (in methanol) showed absorption at 202 nm, along with the characteristic dual absorption bands of isoflavones: band I, contributed by the cinnamoyl conjugation system of ring B, was located at 302 nm (within the 300–400 nm range), and band II, contributed by the benzoyl system of ring A, was located at 249 nm (within the 240–280 nm range). Band I appeared as a shoulder peak with weaker absorption intensity than band II, which is highly consistent with the UV spectral characteristics of isoflavonoids. Infrared spectroscopy analysis showed a broad peak at 3433.99 cm^{-1}, characteristic of O–H stretching vibration, confirming the presence of hydroxyl groups in the molecule. Multiple signals in the range of 2838.02–3134.40 cm^{-1} were attributed to aromatic and aliphatic C–H stretching vibrations. A strong absorption peak at 1734.43 cm^{-1} corresponded to carbonyl (C=O) stretching, indicating the presence of a ketone carbonyl group. Signals in the ranges of 1453.48–1636.35 cm^{-1}, 1026.46–1385.24 cm^{-1}, and 407.23–969.45 cm^{-1} were assigned to aromatic C=C skeletal vibrations, C–O stretching vibrations, and out-of-plane C–H bending vibrations of the aromatic ring, respectively, further supporting the structural features of hydroxyl, carbonyl, and aromatic ring in the molecule. The HPLC chromatogram displayed a single sharp peak, indicating good purity of the compound and meeting the requirements for structural identification. In the negative ion mode mass spectrum, the main deprotonated molecular ion peak was observed at *m/z* 265.0952 ($[M-H]^-$), from which a relative molecular mass of 266.10 was calculated. Software fitting gave the molecular formula $C_{16}H_{12}O_4$, which, together with literature data comparison, preliminarily indicated formononetin. The characteristic fragment ions in the spectrum could be attributed to the loss of neutral small molecules, cleavage of the methoxy group, and skeletal fragmentation products of the isoflavone nucleus, respectively. ^1H NMR spectroscopy showed multiple peaks in the range of δ 6.85–7.97, corresponding to the aromatic protons of rings A and B, and a signal at δ 3.77 characteristic of methoxy protons. In the ^{13}C NMR spectrum, signals in the range of δ 102.14–162.59 covered the aromatic carbons of rings A and B, while signals at δ 55.16 and 174.65 were assigned to the methoxy carbon and the characteristic carbonyl carbon of the isoflavone, respectively. All the above spectroscopic data were consistent with the structural features of formononetin. Regarding the separation methodology, Jiang Shaohua [84] previously used Sephadex LH-20 gradient elution to directly isolate flavonoid monomers. However, direct one-step isolation could not be achieved in this experiment, presumably due to differences in the raw material matrix: the raw material of *L. lucidus* var. *hirtus* contains large amounts of polysaccharides and proteins that are difficult to completely remove, which readily leads to incomplete elution and severe peak tailing during gradient elution, thus preventing the direct isolation of high-purity monomeric components. In this study, by optimizing the separation process, formononetin monomer was successfully isolated from the purified total flavonoids, and its structure was confirmed through the above comprehensive multi-spectroscopic analysis.

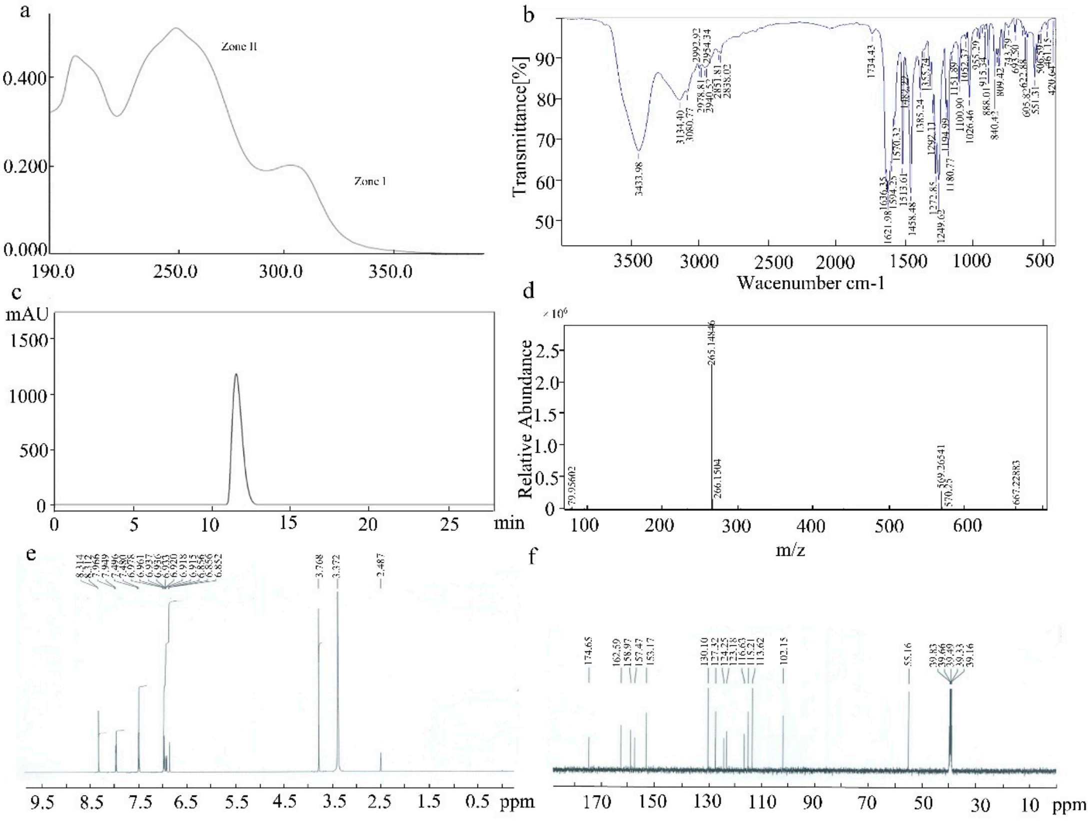

Figure S1. Structural characterization spectra of the isolated flavonoid monomer. (a) UV absorption spectrum; (b) IR absorption spectrum; (c) HPLC chromatogram; (d) Mass spectrometry ion chromatogram;(e) ^{1}H NMR spectrum; (f) ^{13}C NMR spectrum.

Table S1. Spectroscopic comparison between isolated compound and literature reference data for formononetin.

Spectral technique	Data obtained in this study	Reported literature value	Remarks / Agreement	Reference
HR-MS (ESI-) precursor ion	-- m/z 267.0664	-- m/z 267.0662	Mass error +0.75 ppm	Ref-1, Ref-2
MS/MS fragment (ESI-)	m/z 252.0438 ---	m/z 252.1 ---	Diagnostic methyl-loss fragment	Ref-2
UV-Vis (MeOH, λmax, nm)	249, 303	249, 303	Isoflavone characteristic absorption	Ref-1

IR (KBr, main bands, cm^{-1})	3435, 1608, 1514, 1283	3435, 1608, 1514, 1283	Major functional-group bands match	Ref-1
^{1}H-NMR (DMSO-d6, δ ppm, key signals)	8.34 (H-2, s), 7.97 (H-5, d), 7.51 (H-2′,6′, d), 6.99 (H-3′,5′, d), 3.79 (-OCH_3, s)	8.34 (H-2, s), 7.97 (H-5, d), 7.51 (H-2′,6′, d), 6.99 (H-3′,5′, d), 3.79 (-OCH_3, s)	Key diagnostic protons consistent	Ref-1
^{13}C-NMR (DMSO-d6, δ ppm, key signals)	153.7 (C-2), 174.7 (C-4, C=O), 161.0 (C-4′), 55.2 (-OCH_3)	153.7 (C-2), 174.7 (C-4, C=O), 161.0 (C-4′), 55.2 (-OCH_3)	Isoflavone skeleton diagnostic carbons match	Ref-1

Ref-1. Pan C, Wang H, Shan H, Lü H. Preparative Isolation and Purification of Calycosin and Formononetin from Astragali Radix using Hydrolytic Extraction Combined with High Speed Countercurrent Chromatography. Journal of Chromatographic Science. 2021;59(5):412-418.

Ref-2. Xie J, Zhang Y, Wang W, Hou J. Identification and Simultaneous Determination of Glycyrrhizin, Formononetin, Glycyrrhetinic Acid, Liquiritin, Isoliquiritigenin, and Licochalcone A in Licorice by LC-MS/MS. Acta Chromatographica. 2014;26(3):507-516.
